# Trauma triage criteria as predictors of severe injury - a Swedish multicenter cohort study

**DOI:** 10.1186/s12873-022-00596-7

**Published:** 2022-03-12

**Authors:** Lina Holmberg, Kevin Mani, Knut Thorbjørnsen, Anders Wanhainen, Håkan Andréasson, Claes Juhlin, Fredrik Linder

**Affiliations:** 1 Department of Surgical Sciences, Vascular Surgery, Uppsala University, Uppsala, Sweden; 2 Centre for Research and Development, Uppsala University, Uppsala, Region Gävleborg Sweden; 3 Department of Surgical Sciences, Colorectal Surgery, Uppsala University, Uppsala, Sweden; 4 Department of Surgical Sciences, Endocrine Surgery, Uppsala University, Uppsala, Sweden

**Keywords:** Trauma triage criteria, Sensitivity, Specificity, Accuracy, Undertriage

## Abstract

**Background:**

Adequate performance of trauma team activation (TTA) criteria is important in order to accurately triage trauma patients. The Swedish National Trauma Triage Criteria (SNTTC) consists of 29 criteria that trigger either a Trauma Alert, the highest level of TTA, or a Trauma Response. This study aimed to evaluate the SNTTC and its accuracy in predicting a severely injured patient in a multicenter setting.

**Methods:**

A cohort study in Sweden involving six trauma receiving hospitals. Data was collected from the Swedish Trauma Registry. Some 626 patients were analyzed with regard to the specific criteria used to initiate the TTA, injury severity with New Injury Severity Score (NISS) and emergency interventions. Sensitivity, specificity, positive predictive value (PPV) and positive likelihood ratio (LR+) of the criteria were calculated, as well as undertriage and overtriage.

**Results:**

All 29 criteria of SNTTC had a sensitivity > 80% for identifying a severely injured patient. The 16 Trauma Alert Criteria had a lower sensitivity of 62.6% but higher LR+ (3.5 vs all criteria 1.4), specificity (82.3 vs 39.1%) and PPV (55.4 vs 37.6%) and the highest accuracy (AUC 0.724). When using only the six physiological criteria, sensitivity (44.8%) and accuracy (AUC 0.690) decreased while LR+ (6.7), specificity (93.3%) and PPV (70.2%) improved.

**Conclusion:**

SNTTC is efficient in identifying severely injured patients. The current set of criteria exhibits the best sensitivity compared to other examined combinations and no additional criterion was found to improve the protocol enough to promote a change.

## Background

**Fig. 1:**
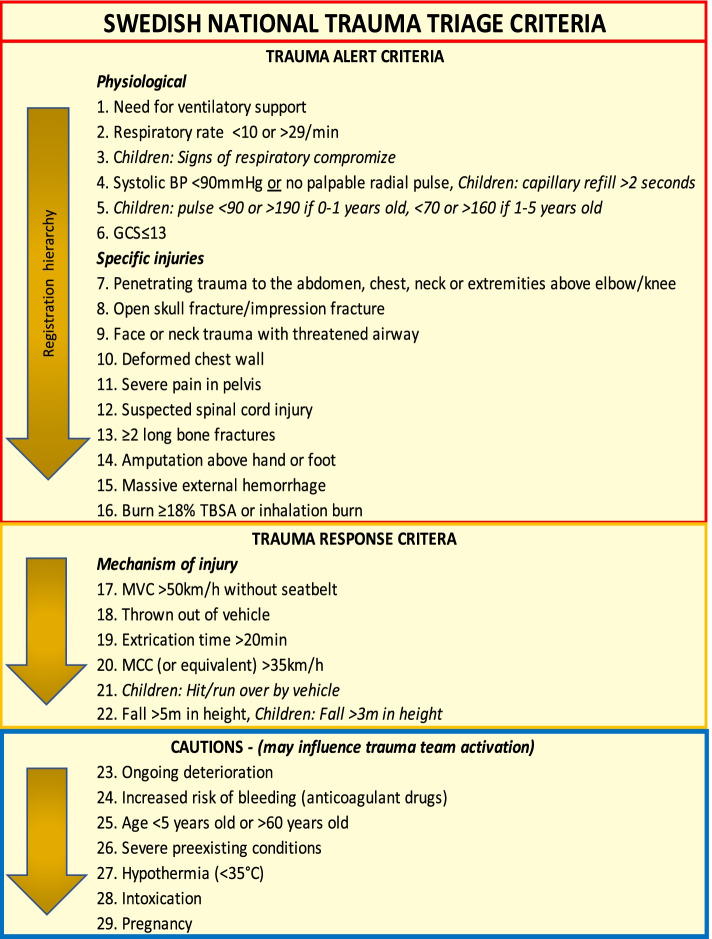
Swedish National Trauma Triage Criteria. BP = Blood pressure, GCS = Glasgow coma scale, TBSA = Total burn surface area, MVC = Motor vehicle crash, MCC = Motorcycle crash, Children = < 15 years old

There has been a number of different trauma team activation (TTA) protocols in Sweden until 2017, when The Swedish National Trauma Triage Criteria (SNTTC, Fig. 1) was implemented after a revision by an expert group assigned by the professional medical societies involved in trauma in Sweden [1]. A study of the SNTTC showed a reduction in the lower-level TTA:s (Trauma Responses) by half, without compromising the undertriage [2], and similar studies evaluating under- and overtriage of TTA protocols exists [3–5]. However, there are few major studies about performance of TTA protocols in-hospital regarding other important performance parameters such as sensitivity, specificity and positive predictive value (PPV). Four single-centre studies [6–9] have evaluated different combinations of sensitivity, specificity and PPV of TTA protocols, but these studies were not comprehensive in terms of trauma population, e.g. excluding pediatric cases or focusing on pediatric or geriatric trauma patients. Evaluation of individual TTA criteria is even more scarce with partly outdated single centre studies; one calculating the odds ratio (OR) for individual criterion [10], another ranking individual TTA criteria using receiver operator characteristics (ROC) to visualize sensitivity, specificity, positive likelihood ratio (LR+) and OR [11] but not calculating sensitivity and specificity of the TTA protocol or reporting PPV for individual criteria.

The need to investigate the performance of TTA criteria, in order to further refine the accuracy in the triage of trauma patients and their correct level of TTA, is therefore paramount. This is especially of interest after implementation of a set of new criteria, such as in the SNTCC situation in Sweden. In this study, we aimed to perform a thorough evaluation of both individual and combinations of TTA criteria in the newly implemented SNTCC in a multicenter setting, including the whole trauma population. The overall objective was to evaluate the SNTTC, individually and in groups, to see if there is a need for further revision of the criteria. The primary aim was to identify the best performing criteria in predicting a severely injured patient and to examine if a combination of criteria could predict the majority of the severely injured patients. Secondary aims were to investigate if there were any criterion that could be omitted in order to enhance the accuracy, and to seek a common denominator among the undertriaged patients that could represent a future trauma triage criterion.

## Methods

### SweTrau and calculating injury severity

Internationally, one of the most commonly used definitions of a severely injured patient has been an Injury Severity Score (ISS) of more than 15 [12]. The modification New Injury Severity Score (NISS) is however regarded as more accurate [13–15] in assessing penetrating trauma victims and in-hospital mortality [14, 16]. NISS> 15 is also one of the inclusion criteria of the Swedish Trauma Registry (SweTrau) [17], founded in 2011, with 92% of the trauma receiving hospitals in Sweden participating in 2018 [18]. In our study, NISS was used to determine the severity of the patient’s injuries.

### Study design

We performed a multicenter cohort study in Mid Sweden involving one trauma center (university hospital) and five trauma receiving hospitals (acute care hospitals), in total serving approximately 950,000 people [19] with a SweTrau registration coverage ratio of 76.9% [18]. Inclusion criterion was all registered patients in SweTrau between 1st of January 2018 and 31st of December 2018. Exclusion criteria consisted of referral patients, patients with missing information about the level of TTA and patients wrongfully registered. The data from SweTrau were supplemented with additional information from the local registrants where data were missing.

### SNTTC

The SNTTC consists of a two-tier system; Trauma Alert is the highest level of TTA and Trauma Response is the lowest (Fig. 1). The criteria for Trauma Alert activation (number 1–16) are arranged in the order of ABCDE with physiological criteria (no. 1–6) and specific injuries from head to toe (no. 7–16), hence the criterion with the lowest number can be expected to first threaten the patients’ life. A Trauma Response (mechanism of injury criteria, no. 17–22) can be upgraded if it is combined with one or more Cautions (no. 23–29), while one or more Cautions might upgrade a non-TTA to a Trauma Response (but not to a Trauma Alert). Severely injured patients (NISS> 15) that do not activate a Trauma Alert are regarded as undertriaged, while patients with NISS< 15 that activate a Trauma Alert are overtriaged, as recommended by the American College of Surgeons Committee on Trauma (ACS-COT) [20].

### Trauma team activating criteria

SweTrau uses variables from the revised Utstein template for uniform reporting of data following major trauma [21]. It does not have a variable defining which criterion that was used to activate the TTA. In order to evaluate each criterion, the participating hospitals used a free variable in SweTrau to record the activating criterion and another free variable for recording if the activated TTA was correct or not, or if a patient who had not activated a TTA should actually have done so. This assessment was done prospectively during the hospitals’ regular trauma registrations in SweTrau. If several criteria had been used, only the criterion with the lowest number was recorded (Fig. 1).

### NISS

NISS is calculated by using the Abbreviated Injury Scale (AIS [22]), a scoring system for trauma patients where a trained registrant scores each injury in different body areas. The patients’ medical charts are reviewed for clinical injuries, radiology reports and operation notes and a total NISS is then calculated in the SweTrau database (AIS version 2005, update 2008).

### Emergency intervention

An emergency intervention is registered in SweTrau if it is performed within 24 h of admission and consists of one or more of the following: thoracotomy, laparotomy, pre-peritoneal pelvic packing, revascularization, endovascular intervention, craniotomy, intracranial pressure device, chest drain, external fixation of fractures, major surgery of fractures, wound revision (in the operating room) or unspecified intervention.

### Statistics

The prevalence of each criterion and combinations of criteria of the SNTTC were examined, together with the percentage of severely injured patients (NISS> 15). The sensitivity, specificity, PPV and LR+ (sensitivity/(1-specificity)) of different combinations of the criteria were calculated, as well as for each criterion with more than five patient activations. Overtriage was determined by the ratio of patients who were not severely injured but had a Trauma Alert according to the criteria, divided by all patients with a Trauma Alert, whilst undertriage equaled patients who *were* severely injured but *did not* have a Trauma Alert according to the criteria, divided by all patients who did not have a Trauma Alert – the Cribari Matrix method [20, 23]. Undertriage was also additionally calculated as the percentage of patients severely injured in total [23]. The accuracy of the criteria was determined by the area under the curve (AUC) from receiver operating characteristics (ROC) -points comparing sensitivity and 1-specificity. The severely injured patients were further analysed to see if there was a difference in characteristics between the undertriaged patients and the patients with a Trauma Alert according to criteria.

Statistical analyses were performed with IBM SPSS Statistics, version 26 (IBM Corp., Armonk, N.Y., USA), with VassarStats, a website for statistical computation (http://vassarstats.net), and with Microsoft Excel for Mac, version 16.16.22. Data were assessed for normality with histograms. Categorical data were analyzed with Chi-square test, except for when the expected count in one cell was < 5 – then Fisher’s exact test was used. Numerical data without normal distribution were assessed with Mann Whitney U test. The level of significance was set at a *p*-value less than 0.05.

## Results

### Study cohort

**Fig. 2:**
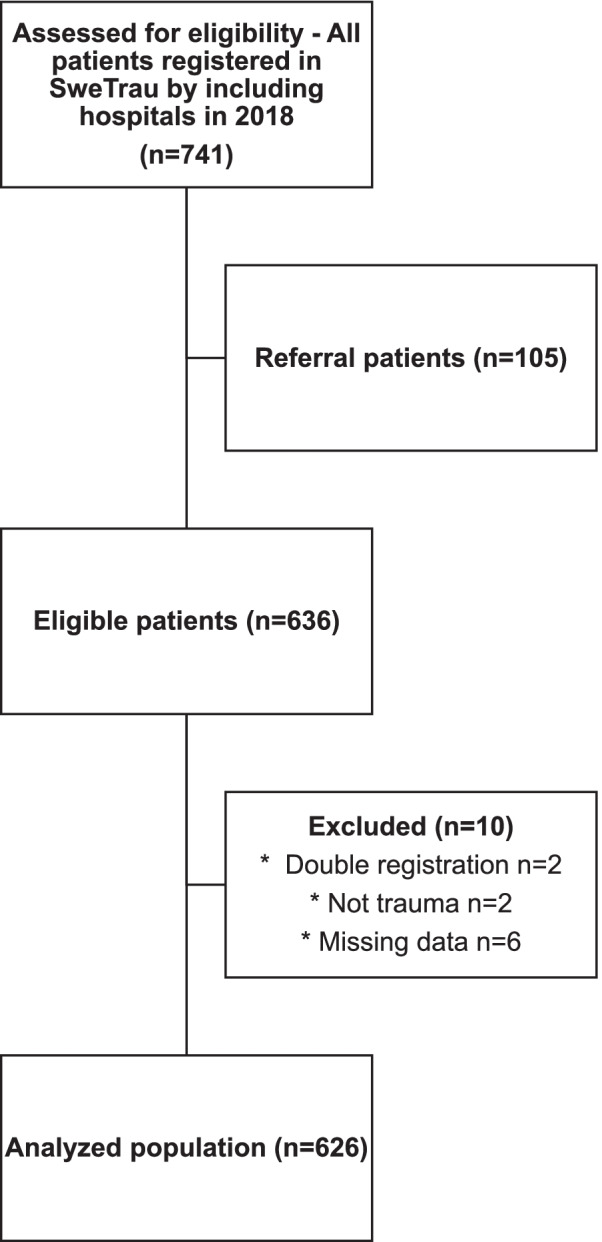
Study patients flowchart, stating reasons for exclusion

A total of 741 patients were identified and after excluding 115 patients (referral patients (*n* = 105), double registrations (*n* = 2), drowning (*n* = 2), missing data (*n* = 6)) a population of 626 patients (Fig. 2) was analyzed with regard to if there had been a TTA according to the SNTTC, if the patient had a NISS> 15 and if the patient had received an emergency intervention. The characteristics of the population and the subgroups of patients with a Trauma Alert, severely injured patients (NISS> 15) and patients requiring an emergency intervention are presented in Table 1. Penetrating trauma represented 9.7% of the population and 11% of the group of NISS> 15, but comprised 25.4% of the Trauma Alert patients and 30.5% of the patients needing an emergency intervention. The two most common mechanisms of injury (MOI); ‘motor vehicle crash, MVC’ (34.7%) and ‘high fall’ (19.3%) made up more than half of the total patients. ‘High fall’ is defined in SweTrau as the patient’s height times 1.5, in coherence with the Utstein Trauma Template – note that this is separate from the Trauma Response criterion no. 22, which is a defined fall height (5 m for adults, 3 m for children) to activate a trauma call.

**Table 1:** Characteristics of study population

Characteristics	Population (***n*** = 626)	Trauma Alert (***n*** = 205)	NISS > 15 (***n*** = 163)	Emergency intervention (***n*** = 95)
Male sex (%)	423 (67.6)	154 (75.1)	127 (77.9)	81 (85.3)
Age – years, median (IQR)	38 (22–57)	35 (22–57)	51 (27–64)	34 (22–56)
Children < 15 years old (%)	56 (8.9)	11 (5.4)	9 (5.5)	5 (5.2)
ASA score 3 or higher (%)	77 (12.3)	28 (13.7)	31 (19.0)	17 (17.9)
NISS – median (IQR)	5 (1–17)	16 (3–27)	25 (18–34)	22 (11–35)
Penetrating trauma (%)	61 (9.7)	52 (25.4)	18 (11.0)	29 (30.5)
Mechanism of injury (%)				
* MVC*	217 (34.7)	49 (23.9)	37 (22.7)	21 (22.1)
* High fall (patient height × 1.5)*	121 (19.3)	38 (18.5)	35 (21.5)	11 (11.6)
* MCC*	79 (12.6)	18 (8.8)	20 (12.3)	13 (13.7)
* Bicycle*	42 (6.7)	13 (6.3)	16 (9.8)	7 (7.4)
* Low fall*	41 (6.5)	9 (4.4)	12 (7.4)	1 (1.1)
* Stabbed with knife/other sharp object*	35 (5.6)	33 (16.1)	7 (4.3)	18 (18.9)
* Hit by blunt object*	26 (4.2)	14 (6.8)	14 (8.6)	6 (6.3)
* Pedestrian*	19 (3.0)	5 (2.4)	5 (3.1)	3 (3.2)
* GSW*	14 (2.2)	13 (6.3)	7 (4.3)	8 (8.4)
* Other vehicle*	11 (1.8)	3 (1.5)	3 (1.8)	1 (1.1)
* Other trauma*	9 (1.4)	3 (1.5)	2 (1.2)	1 (1.1)
* Unknown cause*	7 (1.1)	3 (1.5)	1 (0.6)	2 (2.1)
* Explosion*	5 (0.8)	4 (2.0)	4 (2.5)	3 (3.2)
Private transport (%)	15 (2.4)	5 (2.4)	7 (4.3)	4 (4.2)
Helicopter transport (%)	66 (10.5)	41 (20.0)	34 (20.9)	27 (28.4)
LOS – days, median (IQR)	2 (1–5)	3 (2–9)	7 (2–14)	7 (3–13)
30 day mortality (%)	26 (4.2)	24 (11.7)	20 (12.3)	13 (13.7)

*IQR* Interquartile range, *ASA score* American Society of Anesthesiologists physical status score, *NISS* New Injury Severity Score, *MVC* Motor vehicle crash, *MCC* Motorcycle, *GSW* Gun shot wound. Other vehicle = eg. ship, plane, jetski, LOS = Length of stay. Penetrating injury = Injury resulting from tissue penetrating by a sharp object (e.g., bullet, knife, spear, glass shards, spike, bomb fragments) – including ‘Stabbed with knife/other sharp object’, ‘GSW’ and some patients with ‘Other trauma’

### Prevalence of the criteria

**Fig. 3:**
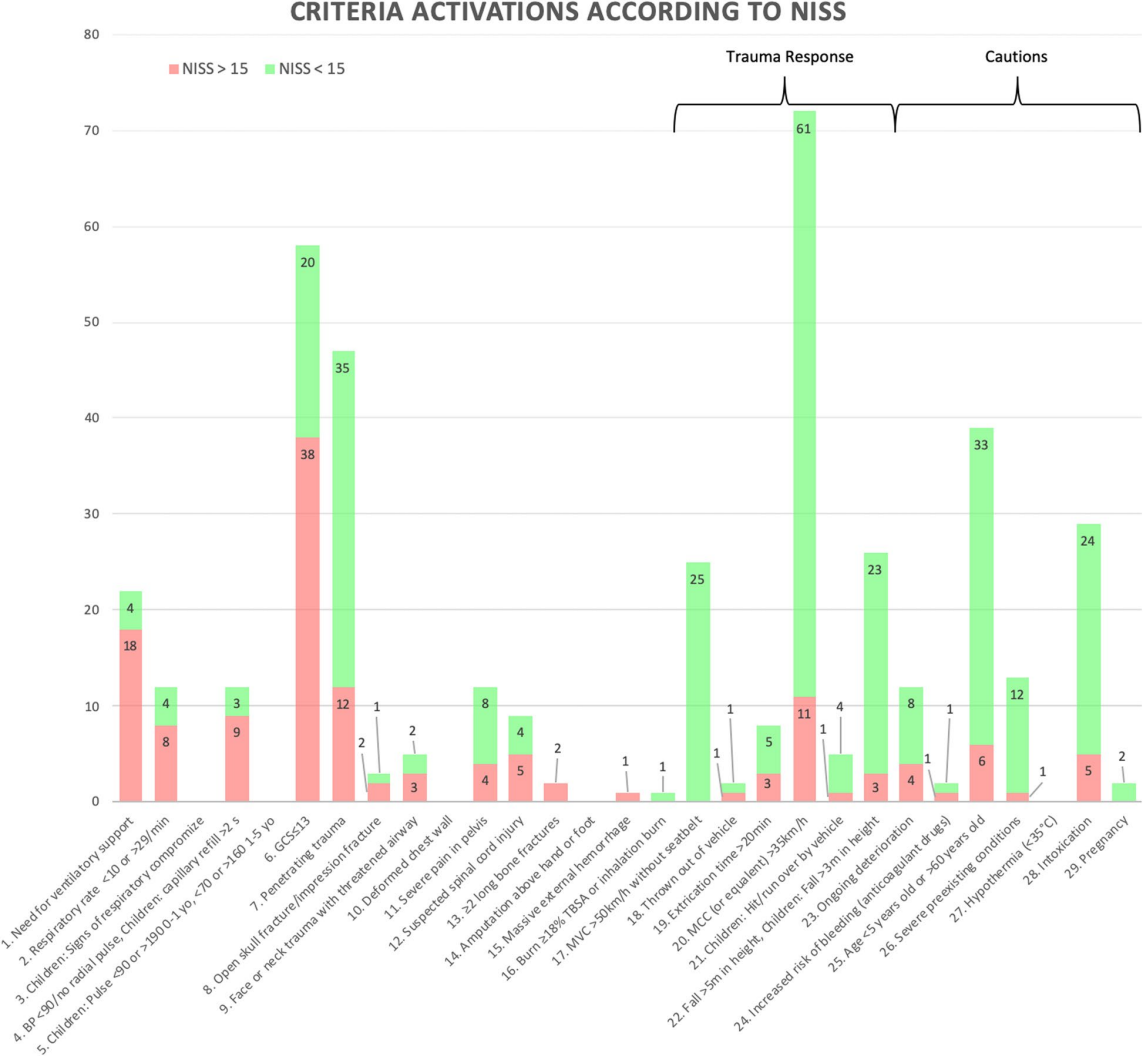
Criteria activations. NISS = New Injury Severity Score. BP = Blood pressure. Yo = Years old. GCS = Glasgow Coma Scale. TBSA = Total burn surface area. MVC = Motor vehicle crash. MCC = Motorcycle crash

The prevalence of criteria activations, grouped by patients severely injured or not, is shown in Fig. 3. The most common criterion was *motorcycle crash > 35 km/h* (MCC, no. 20 in Fig. 1) with 72 activations, of which eleven patients were severely injured. The second most common criterion was *Glasgow Coma Scale < 13* (GCS < 13, no. 6) with 58, whereof 38 had a NISS> 15, followed by *penetrating trauma above elbow or knee* (no. 7) with 47 activations and 12 severely injured patients. Criterion no. 17 (*MVC >  50 km/h without seatbelt*) was activated 25 times but none of the patients were severely injured.

### Accuracy of the criteria

**Fig. 4:**
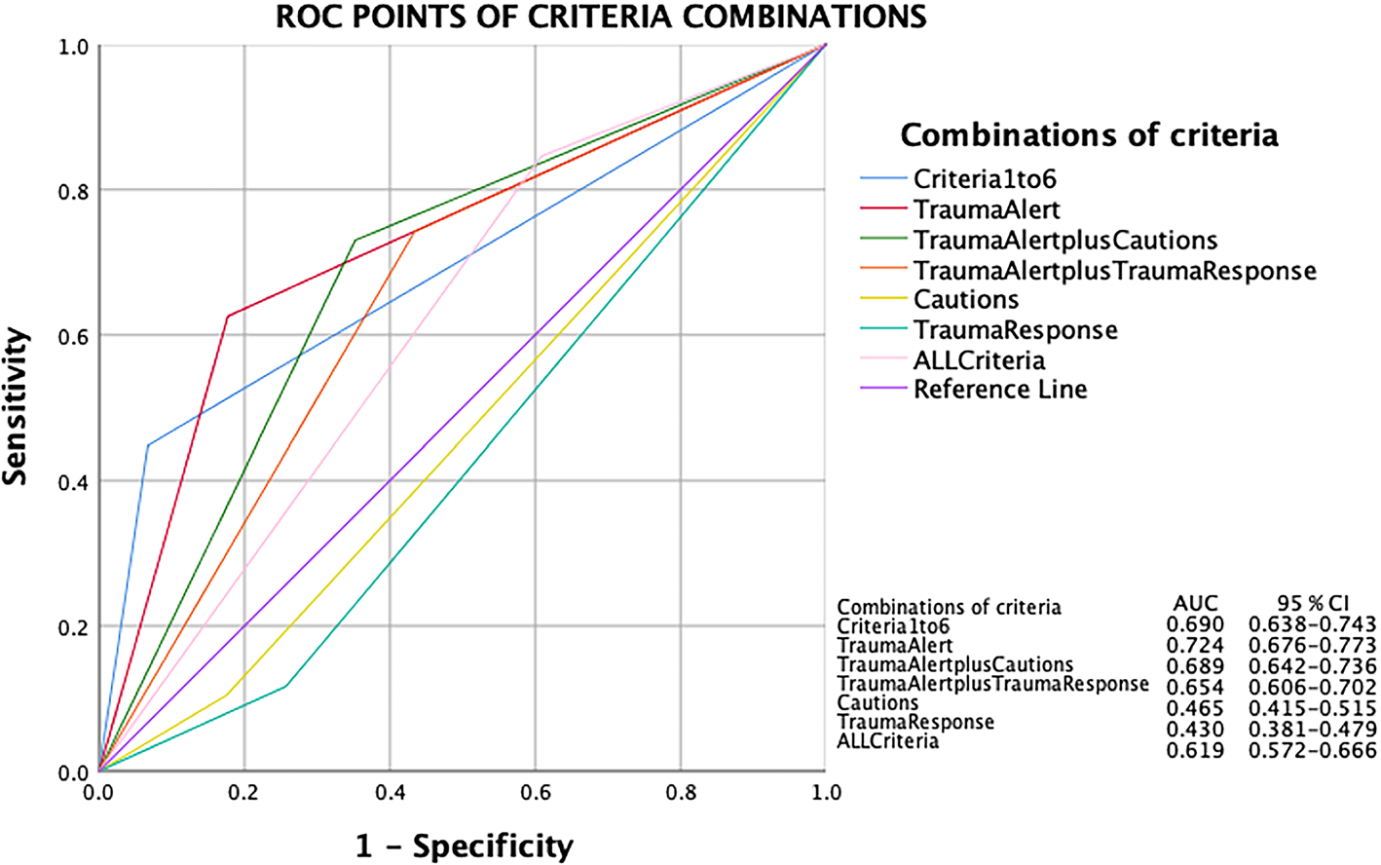
ROC points of criteria combinations

LR+, sensitivity, specificity and PPV of the different criteria combinations and individual criterion with more than five activations are displayed in Table 2. Physiological criteria alone (no. 1–6) had high LR+ (6.7), specificity (93.3%) and PPV (70.2%) but low sensitivity (44.8%). Trauma Alert criteria (no. 1–16) had lower LR+ (3.5), specificity (82.3%) and PPV (55.4%) but higher sensitivity (62.6%). The overall accuracy of the different combinations of the criteria can be visualized in Fig. 4. The combined Trauma Alert criteria had the highest accuracy with an AUC of 0.724, followed by physiological criteria (no. 1–6, AUC 0.690). Cautions and Trauma Response criteria had both an AUC below 0.5 (0.465 and 0.430).

**Table 2:** Criteria statistics

Criteria/criterion		LR + (95% CI)	Sensitivity % (95% CI)	Specificity % (95% CI)	PPV % (95% CI)
Physiological (no. 1–6)		6.7 (4.6–9.8)	44.8 (37.1–52.8)	93.3 (90.5–95.3)	70.2 (60.3–78.6)
Trauma Alert (no. 1–16)		3.5 (2.8–4.4)	62.6 (54.6–69.9)	82.3 (78.4–85.6)	55.4 (47.9–62.7)
Trauma Alert + Cautions (no. 1–16 + 23–29)		2.1 (1.8–2.4)	73.0 (65.4–79.5)	64.8 (60.2–69.1)	42.2 (36.4–48.2)
Trauma Alert + Trauma Response (no. 1–22)		1.7 (1.5–2.0)	74.2 (66.7–80.6)	56.6 (51.9–61.1)	37.6 (32.3–43.1)
All Criteria		1.4 (1.3–1.5)	84.7 (78.0–89.6)	39.1 (34.7–43.7)	32.9 (28.4–37.6)
Cautions (no. 23–29)		0.6 (0.4–1.0)	10.4 (6.4–16.4)	82.5 (78.7–85.8)	17.3 (10.7–26.6)
Trauma Response (no. 17–22)		0.5 (0.3–0.7)	11.7 (7.3–17.8)	74.3 (70.0–78.2)	13.8 (8.7–20.9)
No.	**Individual Trauma Alert** **(> 5 patient activations)**				
1.	Need for ventilatory support	12.8 (4.4–37.2)	11.0 (6.9–17.1)	99.1 (97.6–99.7)	81.8 (59.0–94.0)
4.	BP < 90/no palpable radial pulse, Children: capillary refill > 2 s	8.5 (2.3–31.1)	5.5 (2.7–10.5)	99.4 (98.0–99.8)	75.0 (42.8–93.3)
2.	Respiratory rate < 10 or > 29/min	5.7 (1.7–18.6)	4.9 (2.3–9.8)	99.1 (97.6–99.7)	66.7 (35.4–88.7)
6.	GCS ≤ 13	5.4 (3.2–9.0)	23.3 (17.2–30.7)	95.7 (93.3–97.3)	65.5 (51.8–77.2)
12.	Suspected spinal cord injury	3.6 (1.0–13.1)	3.1 (1.1–7.4)	99.1 (97.6–99.7)	55.6 (22.7–84.7)
11.	Severe pain in pelvis	1.4 (0.4–4.7)	2.5 (0.8–6.6)	98.3 (96.5–99.2)	33.3 (11.3–64.6)
7.	Penetrating trauma	1.0 (0.5–1.8)	7.4 (4.0–12.8)	92.4 (89.5–94.6)	25.5 (14.4–40.6)
No.	**Individual Trauma Response** **(> 5 patient activations)**				
19.	Extrication time > 20 min	1.7 (0.4–7.1)	1.8 (0.5–5.7)	98.9 (97.3–99.6)	37.5 (10.2–74.1)
20.	MCC (or equivalent) > 35 km/h	0.5 (0.3–0.9)	6.7 (3.6–12.1)	86.8 (83.3–89.7)	15.3 (8.2–26.1)
22.	Fall > 5 m in height, Children: Fall > 3 m in height	0.4 (0.1–1.2)	1.8 (0.5–5.7)	95.0 (92.5–96.8)	11.5 (3.0–31.3)
17.	MVC > 50 km/h without seatbelt	NA	0.0 (0.0–0.2)	94.6 (92.0–96.4)	0.0 (0.0–0.2)
No.	**Individual Cautions** **(> 5 patient activations)**				
23.	Ongoing deterioration	1.4 (0.4–4.7)	2.5 (0.8–6.6)	98.3 (96.5–99.2)	33.3 (11.3–64.6)
28.	Intoxication	0.6 (0.2–1.5)	3.1 (1.1–7.4)	94.8 (92.3–96.6)	17.2 (6.5–36.5)
25.	Age < 5 years old or > 60 years old	0.5 (0.2–1.2)	3.7 (1.5–8.2)	92.9 (90.0–95.0)	15.4 (6.4–31.2)
26.	Severe preexisting conditions	0.2 (0.0–1.8)	0.6 (0.0–3.9)	97.4 (95.4–98.6)	7.7 (0.4–37.9)

*LR+* positive likelihood ratio, *PPV* positive predicitive value, *BP* Blood pressure, *GCS* Glasgow Coma Scale, *MCC* Motorcycle crash, *MVC* Motor vehicle crash, *NA* Not applicable

### Overtriage

Overtriage with the Matrix method was 49.8% (102 of 205 patients). Twenty-one percent of the overtriaged patients needed an emergency intervention (21/102). Of the overtriaged patients, 32 (31.4%) activated criteria no. 7 (*penetrating trauma above elbow or knee*) of which 14 (43.8%) needed an emergency intervention.

### Undertriage

Undertriage with the Matrix method was 11.2% (47 of 421 patients) while the undertriage was 28.8% (47/163) of the severely injured patients in total. Half of the undertriaged patients (24 patients) activated a Trauma Response or Cautions and the rest did not activate a criterion. Fifty percent of the patients that activated a criterion triggered either no. 20 (*MCC*, six patients), or criterion no. 25 (*Age < 5 or > 60*, six patients). Of 72 activations of no. 20, only 7 patients were > 60 years old, but 5 of those had an NISS> 15. The majority of undertriaged patients that did not activate a criterion (17/23) had either fallen less than 5 m or been hit by a blunt object. When comparing the undertriaged patients with the correctly triaged patients with NISS> 15 (Table 3), there was a higher percentage of children (12.8% vs. 2.6%, *p* = 0.018) and a higher ASA score (30.4% vs. 15.2%, *p* = 0.028) among the undertriaged patients. The NISS was lower (19 vs. 27, *p* = 0.000), there was no penetrating trauma (0% vs. 15.5%, *p* = 0.004) and there was a less need for emergency intervention (10.6% vs. 50.9%, *p* = 0.000). One of the undertriage patients died within 30 days, compared to 19 of the correctly triaged patients (2.2% vs. 17.1%, *p* = 0.011).

**Table 3:** Severely injured patients – differences between patients with Trauma Alert (according to criteria) and undertriaged patients (NISS> 15 but no Trauma Alert according to criteria)

Characteristics of severely injured patients (NISS > 15, *n* = 163)	Trauma Alert (***n*** = 116)	Undertriage (***n*** = 47)	***P***-value
Age - years, median (IQR)	48.5 (27–61)	55 (29–69)	0.259^2^
Children < 15 years old (%)	3 (2.6)	6 (12.8)	0.018*^3^
ASA score 3 or higher (%)	17 (15.2)	14 (30.4)	0.028*^1^
NISS - median (IQR)	27 (22–42.5)	19 (17–24)	0.000*^2^
Penetrating trauma (%)	18 (15.5)	0 (0)	0.004*^1^
Emergency intervention (%)	59 (50.9)	5 (10.6)	0.000*^1^
LOS - median (IQR)	8 (2–15)	6 (3–11)	0.418^2^
30 day mortality (%)	19 (17.1)	1 (2.2)	0.011*^1^

*NISS* New Injury Severity Score, *IQR* Interquartile range, *ASA score* American Society of Anesthesiologists physical status score, *LOS* Length of stay. ^1^Chi-Squared test. ^2^Mann-Whitney U test. ^3^Fisher’s exact test. * = *p* < 0.05

## Discussion

This multicenter evaluation of the newly implemented SNTTC confirms that the current combination of the 29 criteria has adequate sensitivity and LR+ to reliably identify severely injured patients, with an AUC of 0.619. Although a selection of a subgroup of the criteria, e.g. physiological criteria alone or the Trauma Alert criteria alone would increase the AUC and specificity, this would occur at a cost of reduced sensitivity. Among undertriaged patients, no specific criteria suitable for inclusion in the trauma triage system could be identified. Notably, one of the drawbacks of the SNTTC is the extensive number of variables. Although the current study could not identify specific variables that were clearly unnecessary as part of the criteria, further refinement of trauma triage criteria to streamline processes with a minimum number of criteria, while upholding adequate sensitivity, is of value for trauma optimization.

### Study cohort

The characteristics of our study population is consistent with other international studies [2–4, 6, 9, 10, 13] with a male predominance, a clear majority of blunt trauma, a young median age at around 38 but with the severely injured patients being older*.* In the Trauma Alert and emergency intervention subgroups, penetrating trauma is the leading cause although only comprising about 10% of the total population. This is explained by the fact that penetrating trauma results in activation of Trauma Alert based on a specific criterion, and penetrating trauma is in itself prone to need emergency intervention. Overall 30 day mortality is in line with other studies [2–4, 6, 9] as is the expected higher mortality of the Trauma Alert group [2–4].

### Prevalence of the criteria

The criteria not used or with < 5 activations could be explained by various factors, including too few patients – especially for no. 16 (*burn ≥ 18% or inhalation burn*) where the low frequency of major burn injuries might need a bigger study cohort. Another reasonable explanation is that many of the patients with a higher number, for example criteria no. 10 (*deformed chest wall*) or no. 6 (*GCS < 13*) could also activate a lower number (e.g. no. 1, *need for ventilatory support*). Unfortunately, since only the lowest number was recorded there is not enough information to suggest removal of the unfrequently used criteria with a higher number.

Criterion no. 20 (*MCC*) was the most prevalent criterion but the majority of patients were not severely injured. Nevertheless, eleven patients had a NISS> 15, why the criterion’s position among the Trauma Response Criteria still seems appropriate. Criterion no. 17 (*MVC*) was activated 25 times without identifying a single severely injured patient which makes it a possible candidate to consider for removal from the SNTTC, even though this must be examined further before any definitive conclusions can be drawn.

### Accuracy of the criteria

The aim is to get a high sensitivity (percentage of patients with NISS> 15 that activate the criteria) but not at the expense of too low specificity (percentage of patients with NISS< 15 that not activate the criteria), which is best illustrated by the accuracy shown by AUC [24] of the different combinations of criteria. In the trauma triage evaluation literature, AUC is not commonly used as a statistical method, and we only found one such study [11], from California. The trauma criteria of this study are quite similar to SNTTC and out of the five SNTTC criteria with the highest LR+ (e.g. the slope of the ROC-curve generating the AUC [25]), four (no. 1, 2, 12 and 4) were found among the top five of the above mentioned study. However, the Californian study does not compare the AUC of different combinations of criteria (which is the case in the current study); which makes further comparisons difficult.

Different studies have chosen different measurements to evaluate and then decide which criteria they believe to be superior. If one considers AUC the best measurement, then the Trauma Alert Criteria (no. 1–16) have the highest predictivity (although too low sensitivity to be acceptable). Sensitivity, specificity and the accuracy reflected by the AUC is more of a measurement of the ‘general strength’ of the criteria, compared to PPV and LR+ that better calculate the ‘clinical usefulness’ of the criteria. PPV shows the specific probability that a patient is severely injured if the criterion/criteria is activated but it is influenced by the prevalence of severely injured patients in the population [26]. LR+, on the other hand, is not sensitive to prevalence [27]. It is interpreted as how many times more likely it is for a severely injured patient to activate the criterion in question than for a not severely injured patient. The higher LR+, the stronger the association with NISS> 15. When dealing with LR+ one must also consider the subjective ‘pretest probability’ of the patient the criterion is applied to, which means that two different patients may have different risks to be severely injured, given a certain criterion with a specific LR+. In our study, this is especially relevant to the Trauma Response criteria where an elderly patient with anticoagulant medication that has been in a motorcycle crash is more likely to be severely injured than a young, healthy patient – even though the LR+ for *MCC* is 0.5 in both cases. The ‘pretest probability’ is in this case highlighted by the two Cautions used: ‘elderly’ (no. 25) and ‘anticoagulant medication’ (no. 24). The SNTTC thus has Cautions as an ‘built-in pretest probability’, making LR+ a good measurement for the usefulness of the criteria. If PPV or LR+ are considered the best tests to find a severely injured patient, then the physiology criteria (no. 1–6) have the highest predictivity, however, they also have too low sensitivity to be acceptable.

Overall, when looking at these statistics, one is tempted to suggest to only use Trauma Alert criteria (highest AUC) or even only use the physiological criteria (no. 1–6, highest LR+ and specificity and PPV). The problem with this reasoning is that the sensitivity of both Trauma Alert criteria (62.6%) and, especially, the physiological criteria (44.8%) are not sufficient, which would lead to unacceptable high undertriage if these two combinations were to be used independently as sole trauma triage criteria. If we consider another scenario; taking the combination ‘Trauma Alert + Cautions’, that interestingly have a better LR+ (2.1 vs. 1.7), specificity (64.8 vs. 56.6%) and PPV (42.2 vs. 37.6%) than ‘Trauma Alert + Trauma Response’, the sensitivity is still only 73%. Considering all this, the only combination with a high enough sensitivity to be acceptable is SNTTC:s current combination of All criteria (84.7%), even though we have to accept lower LR+ (1.4), specificity (39.1%) and PPV (32.9%).

Although the local protocol of trauma triage criteria from a previous study [6] at a Swedish trauma center appears to have higher sensitivity (90.3% vs 84.7%) and specificity (48.2% vs 39.1%) than SNTTC, the confidence intervals of sensitivity overlap and, more importantly, nearly 30% of the included patients in the study had missing data which makes the results not comparable. In a Norwegian study [9] the sensitivity of the trauma triage criteria was 87%, but again with overlapping confidence intervals with SNTTC and with a significantly lower PPV (22% (CI 20–26) vs SNTTC (39% (CI 35–44)). PPV of SNTTC:s Trauma Alert criteria alone is 55.4% compared to 51.7% in an American study [11], indicating that SNTTC is performing well in an international comparison.

### Overtriage

The overtriage with the Matrix method is nearly 50%, similar to the overtriage in two studies from Norway (55%) [28] and the USA (45%) [29]. This is higher than the 25–35% the ACS-COT recommends [20], but a clear improvement since the implementation of the SNTTC in 2017 when the overtriage was calculated to 72.2% [2]. Penetrating trauma was more than three times as common among the overtriaged patients than in the total population, showing that a single stab– or gunshot wound not necessarily generates a NISS > 15 and thus; that the Trauma Alert criteria not only identifies the severely injured patients, but also the patients in need of an emergency intervention. It is important to acknowledge that NISS is only known after discharge, and hence there is clearly a need for triage criteria to prudently identify patients at risk of developing major complications or needing acute interventions. In the current study, the overtriaged patients contributed to more than a fifth (22.1%) of the total emergency interventions. The overtriage should therefore be considered with this in mind.

### Undertriage

Compared to a previous study in the mid Sweden region [2] the undertriage with the Matrix method have increased from 7.1 to 11.2%, which is higher than the 5% recommended by ACS-COT [20], but comparable with a study from Norway [28] (10% undertriage) and considerably lower than in a study from the USA [29] (24% undertriage). This increase can partly be explained by the decrease in number of Trauma Responses/No trauma activations (79.3% vs 67.3%) [2] and since this means a decrease in the denominator it will lead to a higher undertriage. Another, and perhaps more appropriate, way to compare the undertriage is to calculate it as percentages of the total number of severely injured patients [23], which on the contrary shows a clear reduction of undertriage from 49.4% (37/79) in the previous study [2] to 28.8% (47/163) in this study.

A closer evaluation of the undertriaged patients unfortunately did not reveal a common denominator to be considered as a future criterion, however, the combination of Trauma Response no. 20 (*MCC*) + Caution no. 25 (*Age > 60*) appears very troublesome, which is a strong indicator that the Cautions indeed should be used together with the Trauma Response criteria to activate a Trauma Alert, and also underlines the well-known problem with compliance to trauma alert criteria [30]. The importance of old age as an TTA criterion, except when it comes to ground-level falls, is equally highlighted in an American study [31] but with the cut-off limit set at 70 years old.

### Limitations and strengths

This study is a multicenter report, covering both a trauma center and trauma receiving hospitals with the same, national trauma triage criteria, which is a strength in terms of generalizability of the results. The SNTTC are to a large extent similar to other international trauma triage criteria, for example Norway’s, although with regional differences of the combinations. As this study evaluates both the individual criteria as well as different combinations, the results could be of value for other trauma systems than the Swedish cohort currently studied.

This study has the general limitations of a retrospective cohort study, depending on the accurate registration of collected data. There may be a patient selection bias due to patients missed and therefore not registered in SweTrau. However, the registrars had received thorough instructions to scan the admissions to all intensive and intermediate care wards for trauma patients. Furthermore, they received lists of all trauma calls from the hospitals’ central pager system and they were also instructed to manually check all trauma admissions to the emergency department during the study period. Thus, the effect of possible missing cases on the outcome of this study should be minimal. The SweTrau data was in some cases supplemented with information from the registrars for completeness, which is a potential source of bias. The registrars were not blinded to the trauma activation criteria when calculating NISS and this could potentially be a limitation to the study. However, as NISS comprises an objective calculation of the specific injuries of the patient according to AIS 2005 rev. 08, this lack of blinding should not result in significant bias. To minimize the potential differences when calculating the AIS and NISS the registrars had completed the international accreditation course for AIS-coding and used the integrated AIS-module in SweTrau based on AIS 2005 rev. 08. There are also some limitations when using the Cribari Matrix method to calculate undertriage, for example; when the Trauma Responses decline, undertriage will increase due to the construction of the calculating formula. We have tried to address this issue by also using an additional method when calculating undertriage, as described in Methods, section *Statistics and ethics*. Finally, there may have been too few patients to properly evaluate some criteria resulting in a risk for type II error, especially since only 163 patients had a NISS> 15, and we encourage future studies to validate our results.

## Conclusions

The SNTTC is efficient in identifying severely injured patients and patients in need of an emergency intervention, with decreased undertriage as well as overtriage since its implementation. The Trauma Alert Criteria (no. 1–16) has the highest accuracy of the criteria combinations, however, none of the combinations have a high enough sensitivity to replace All criteria. An analysis of the undertriaged patients did not identify any additional criteria to add to the SNTTC in order to further reduce undertriage. Since there is a risk that our study may have too few patients to make more far-reaching conclusions, the authors strongly suggest that trauma registries include the triage criterion used to trigger the trauma call as a variable to continue to evaluate trauma triage criteria. We would also like to underline the necessity of all trauma receiving hospitals to register in trauma registries.

## Data Availability

Data available from the corresponding author upon reasonable request.

## Abbreviations

ACS-COT: American College of Surgeons Committee on Trauma
AIS: Abbreviated Injury Scale
AUC: Area under the curve
GCS: Glasgow Coma Scale
ISS: Injury Severity Score
LR+: likelihood ratio
MCC: Motorcycle crash
MOI: Mechanisms of injury
MVC: Motor vehicle crash
NISS: New Injury Severity Score
OR: Odds ratio
PPV: Positive predictive value
ROC: Receiver operator characteristics
SNTTC: Swedish National trauma Triage Criteria
SweTrau: Swedish Trauma Registry
TTA: Trauma Team Activation
